# Case Report: Response to Intra-arterial Cisplatin and Concurrent Radiotherapy Followed by Salvage Surgery in a Patient With Advanced Primary Sinonasal Low-Grade Non-intestinal Adenocarcinoma

**DOI:** 10.3389/fsurg.2020.599392

**Published:** 2020-12-10

**Authors:** Hirohiko Tachino, Hiromasa Takakura, Hideo Shojaku, Michiro Fujisaka, Katsuichi Akaogi, Hideto Kawabe, Norihito Naruto, Hiroko Shojaku, Kyo Noguchi, Shigeharu Miwa, Johji Imura, Yoshinobu Maeda

**Affiliations:** ^1^Department of Otolaryngology, University of Toyama, Toyama, Japan; ^2^Department of Otolaryngology, Toyama Red Cross Hospital, Toyama, Japan; ^3^Department of Radiology, University of Toyama, Toyama, Japan; ^4^Department of Diagnostic Pathology, University of Toyama, Toyama, Japan; ^5^Department of Pathological Diagnosis, Toyama Red Cross Hospital, Toyama, Japan

**Keywords:** sinonasal adenocarcinoma, low grade, non-intestinal type, T4a, oncurrent chemoradiotherapy, intra-arterial cisplatin, complete remission

## Abstract

**Background:** The clinical usefulness of concurrent chemoradiotherapy before surgery in the treatment of primary, locally advanced sinonasal low-grade, non-intestinal type adenocarcinoma (LG non-ITAC) is unclear.

**Methods:** We present the first case report of the efficacy of super-selective intra-arterial cisplatin (CDDP) infusion concurrent with conventional fractionated radiotherapy (RT) for LG non-ITAC in a Japanese patient.

**Results:** A white, rugged-marginal mass that was histopathologically diagnosed as LG non-ITAC occupied the right nasal cavity. Based on the imaging findings, including computed tomography, magnetic resonance imaging, and positron emission tomography, the tumor was diagnosed as T4aN0M0, stage IVa. After treatment, the nasal tumor disappeared leaving only a small bulge in the medial wall of the middle turbinate. The patient also underwent right transnasal ethmoidectomy performed as salvage surgery. A histopathological examination revealed that the lesion was replaced by granulation tissue with lymphocytic infiltration and hemosiderin-laden macrophages, and no viable tumor cells remained. In the seven years after treatment, the patient has not experienced any local recurrence or regional or distant metastasis.

**Conclusions:** Super-selective intra-arterial CDDP infusion concurrent with conventional fractionated RT *followed by salvage surgery* might be useful for the management of sinonasal LG non-ITAC.

## Introduction

In Japan, the frequency of the sinonasal cancer among patients with head and neck cancer is approximately 8% (1). The most common histopathological type is squamous cell carcinoma (56.8%), then malignant melanoma (11.2%), olfactory neuroblastoma (6.1%), and adenoid cystic carcinoma (5.6%) in the nasal cavity and paranasal sinus. The frequency of sinonasal adenocarcinoma among sinonasal carcinomas in Western countries is 10~50%, which is relatively high in comparison to the frequency in Japan (2.3%) (2–4). Based on the WHO classification in 2017, sinonasal adenocarcinoma is categorized into intestinal and non-intestinal types (5). In contrast to intestinal-type adenocarcinoma (ITAC), which is associated with wood and leather dust exposure, non-intestinal type adenocarcinoma (non-ITAC) is not associated with any environmental factors (6). Non-ITAC is further classified into low-grade (LG) non-ITAC and high-grade (HG) non-ITAC (5, 6).

Many authors consider that surgery followed by radiotherapy represents the gold standard in the management of sinonasal adenocarcinoma (5, 7, 8). To improve local control, preserve organ function and prevent disfigurement, some centers have explored adding chemotherapy to standard treatment (4, 9). Recently, the usefulness of chemotherapy concurrent with radiotherapy in squamous cell carcinomas and malignant melanoma of the sinonasal area has been demonstrated (10, 11). Little is known about the usefulness of concurrent chemoradiotherapy in sinonasal LG non-ITAC. Herein, we report the case of a patient with primary, locally advanced sinonasal LG non-ITAC that was completely treated with intra-arterial cisplatin (CDDP) concurrent with radiotherapy *followed by salvage surgery*. Written informed consent was obtained from the patient for the publication of any potentially identifiable images or data included in this article.

## Case Report

**Figure 1:**
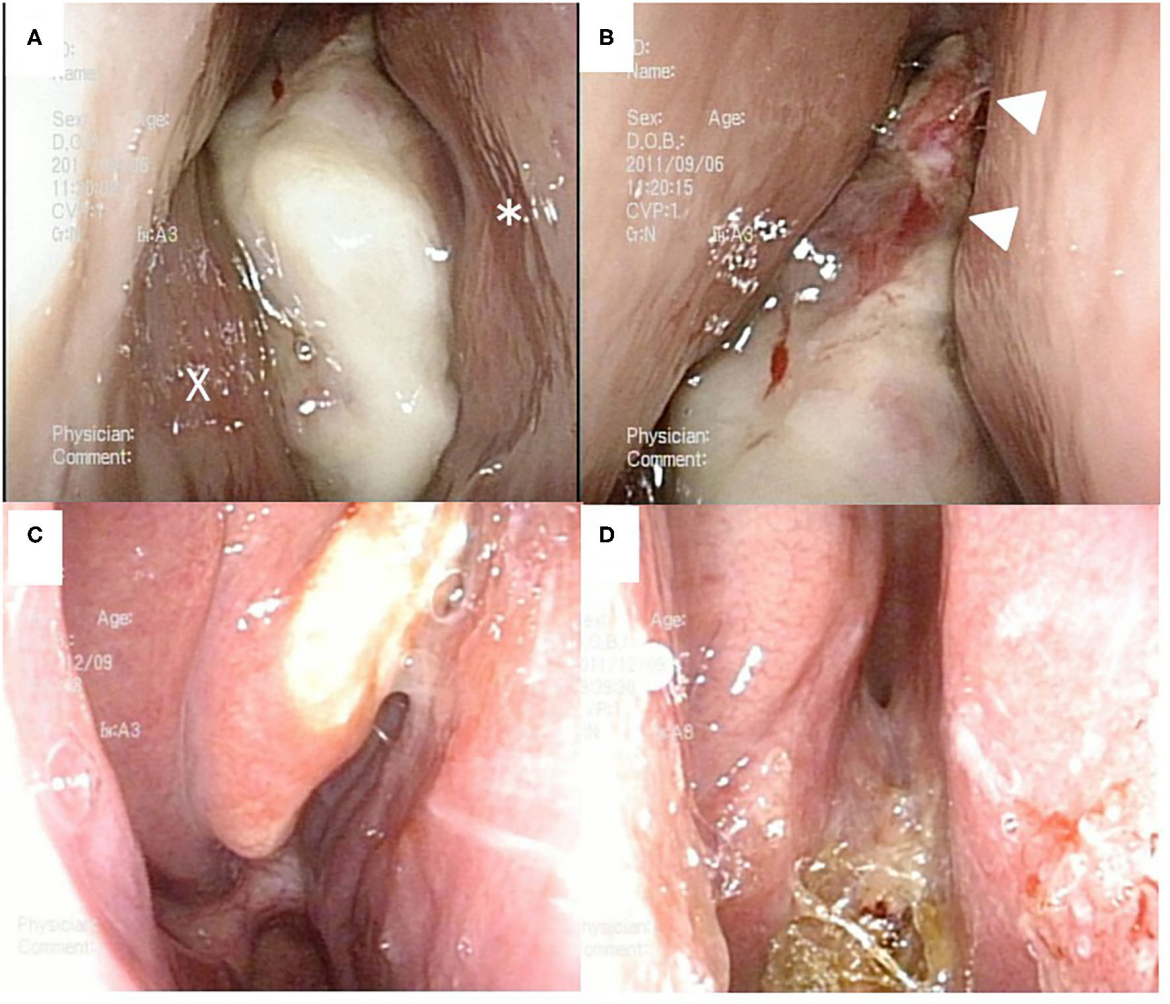
Nasal endoscopic findings. Before chemoradiotherapy (**A,B**). A white tumor occupied right nasal cavity. X and * indicate the inferior nasal turbinate and nasal septum, respectively. After chemoradiotherapy (**C,D**). A white tumor was localized at the medial surface of right middle nasal turbinate (**C**). Right common nasal meatus was widely opened, and the olfactory cleft was clearly visualized (**D**).

**Figure 2:**
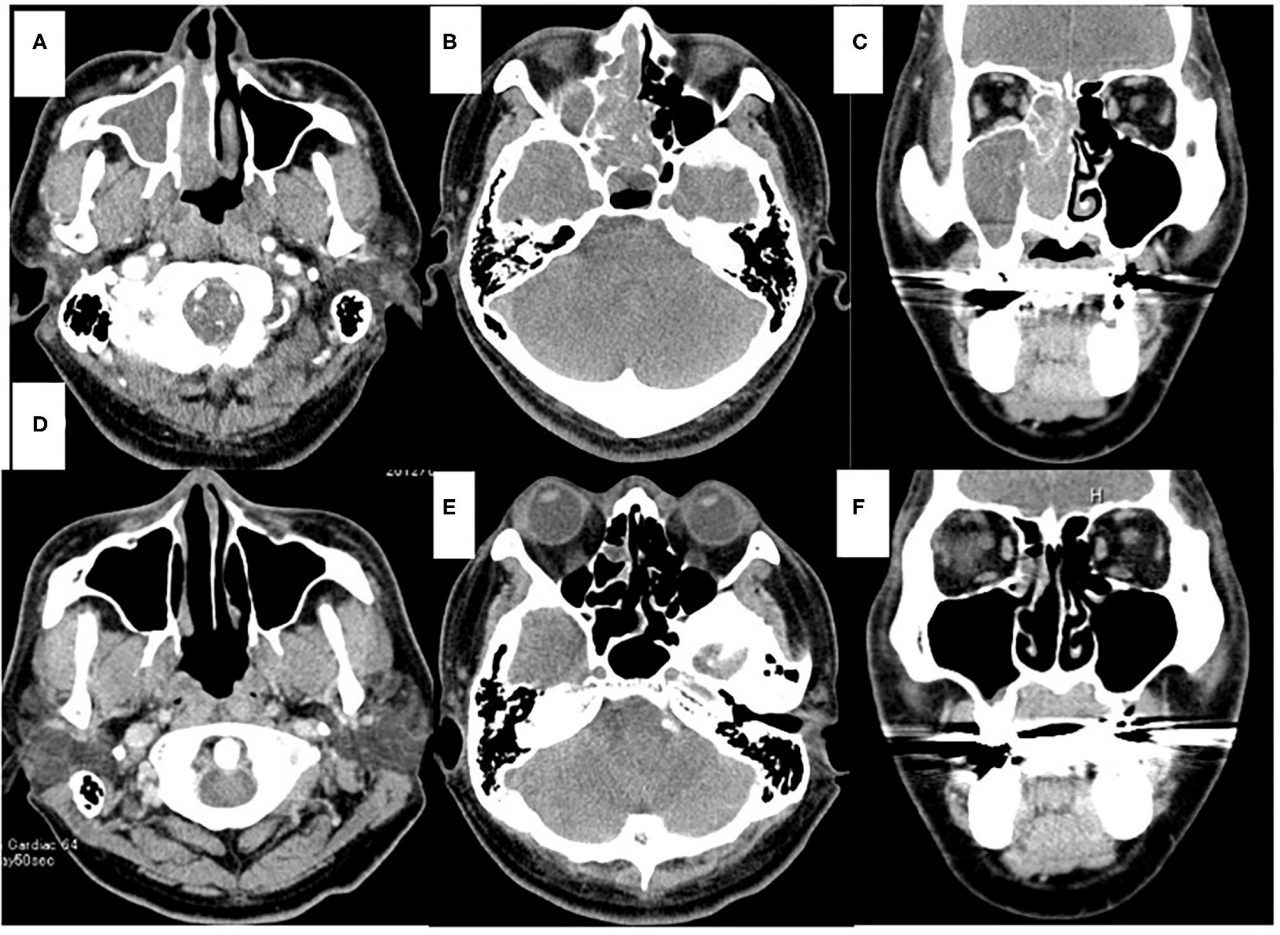
Computed tomography (CT) of the sinonasal region. Axial view (**A,B,D,E**). Coronal view (**C,F**). Enhanced CT scans obtained before chemoradiotherapy (**A–C**). On the right side, the nasal cavity and paranasal sinus were fully opacified. On the left side, the part of the sphenoid sinus were opacified. CT scans obtained after chemoradiotherapy (**D–F**). Opacification was only localized in the part of the right ethmoid sinus (**E,F**).

**Figure 3:**
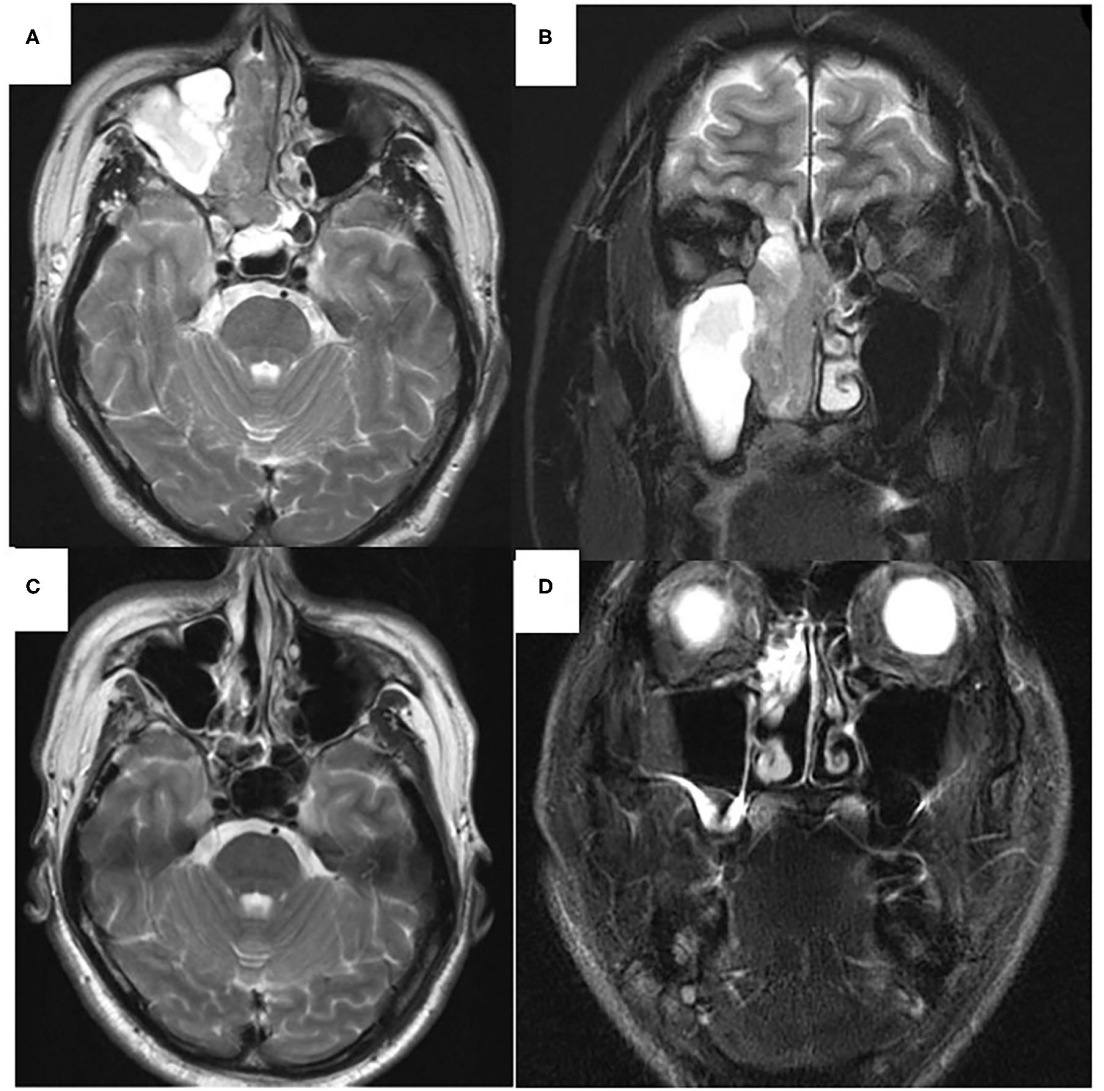
T2-weighted magnetic resonance imaging (MRI) of the sinonasal region. Axial view (**A,C**). Coronal view (**B,D**). Before chemoradiotherapy (**A,B**). Low signal area was observed mainly in the right nasal cavity, ethmoid and sphenoid sinuses. High signal intensity area was found in right maxillary and left sphenoid sinuses. After chemoradiotherapy (**C,D**). In right maxillary sinus, high signal intensity area was disappeared (**C**). High signal intensity area was observed within the ethmoid sinus (**D**).

**Figure 4:**
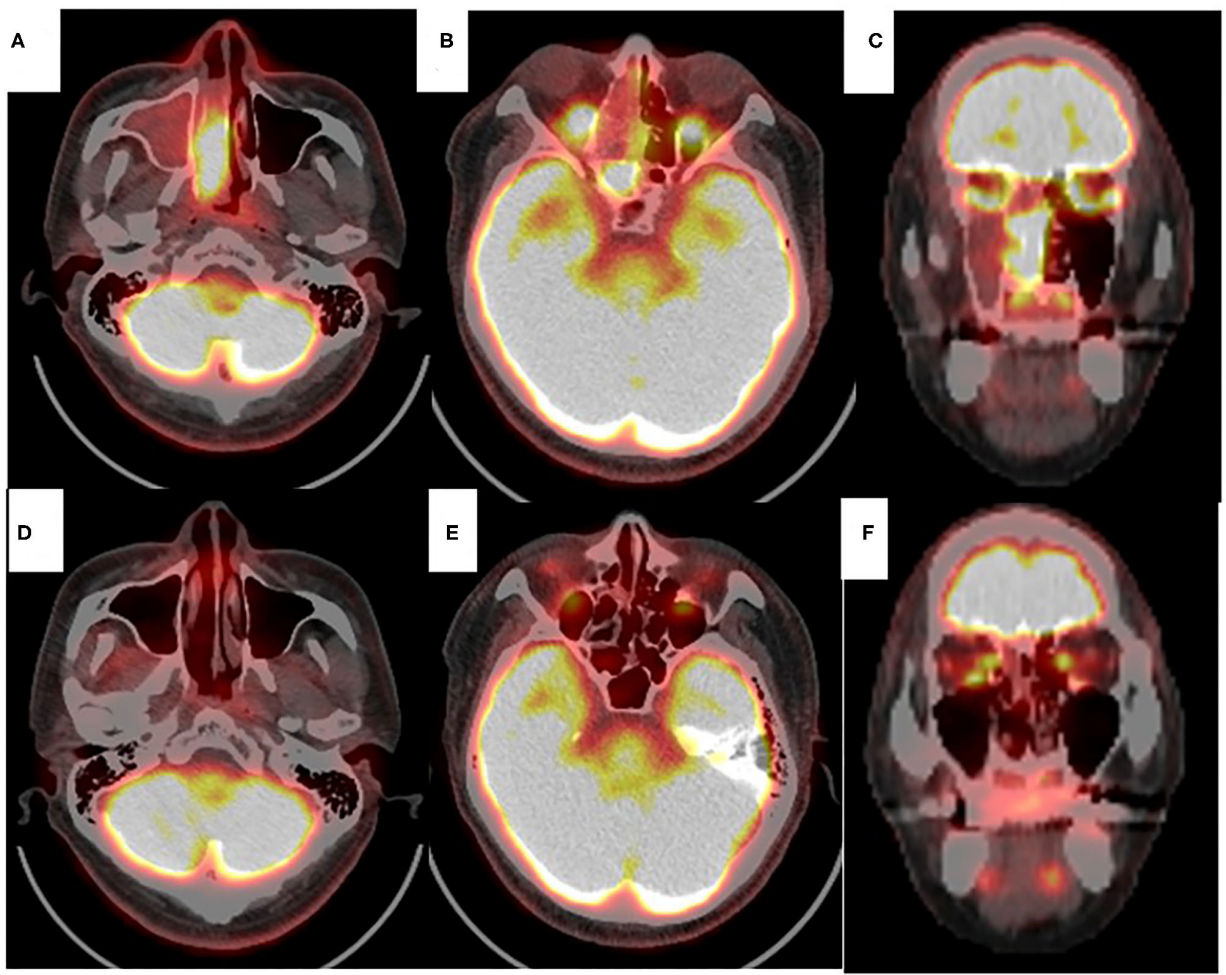
Positron emission tomography with 18F-fluorodeoxyglocose (FDG PET) of the sinonasal region. Axial view (**A,B,D,E**). Coronal view (**C,F**). Before chemoradiotherapy (**A–C**). Significant FDG uptake was found in right nasal cavity, ethmoid and sphenoid sinus. PET scans obtained after chemoradiotherapy (**D–F**). No significant FDG uptake was found in right nasal cavity or paranasal sinus (**D–F**).

**Figure 5:**
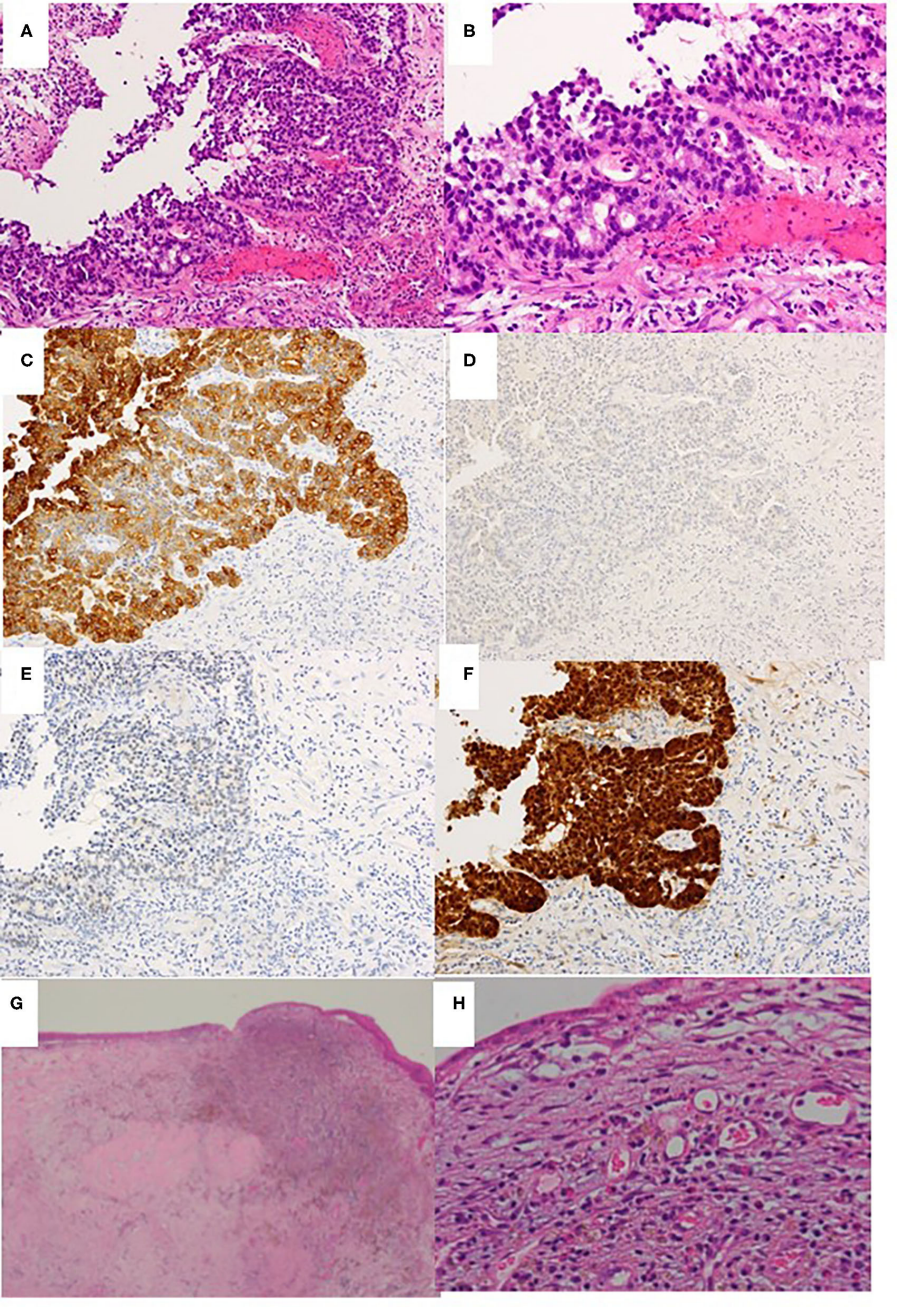
Histopathological examination of biopsy specimen obtained before chemoradiotherapy **(A–F)** and the excised middle meatus after chemoradiotherapy **(G,H)**. **(A)** Hematoxylin & eosin staining (H&E) ×40. **(B)** H&E ×100. **(C)** Cytokeratin 7 immunostaining ×40. **(D)** Cytokeratin 20 immunostaining ×40. **(E)** CDX2 immunostaining ×40. **(F)** P16 immunostaining ×40. **(G,H)** H&E ×40 and ×100, respectively.

A 41-year-old man presented to the otolaryngological department of another hospital with a 12-month history of right nasal obstruction, epistaxis and periorbital pain. Because the nasal tumor was found in the right nasal cavity, he was referred to our university hospital. Nasal endoscopy revealed a white tumor with a rugged margin that occupied the right nasal cavity (Figures 1A,B). The color of the tumor in the uppermost part of the right nasal cavity was slightly pink. There was no regional lymphadenopathy. Sinonasal contrast computed tomography (CT) confirmed the presence of partially enhanced opacification in the right nasal cavity (Figures 2A–C). In addition, homogenous opacification was found in the right frontal, maxillary, ethmoidal, and sphenoid sinuses, and a potential bone defect was found in the medial wall of the maxillary sinus and the uppermost part of the nasal septum. On the left side, the uppermost part of the nasal cavity and anterior part of the sphenoid sinus were opacified. The posterior part of the sphenoid sinus was not opacified, suggesting that the tumor extended into the sphenoid sinus. T2-weighted magnetic resonance imaging (MRI) showed a right sinonasal mass with a low signal intensity, which mainly occupied the nasal cavity and part of the ethmoid sinus (Figures 3A,B). An area of high signal intensity was found in the right frontal, maxillary, sphenoid, and remaining ethmoid sinus. On the left side, the uppermost part of the nasal cavity showed a low signal intensity, and the anterior part of the sphenoid sinus showed a high signal intensity. 18F-Fluorodeoxyglucose-positron emission tomography (FDG-PET) showed significant FDG uptake in the right nasal cavity and the ethmoid and sphenoid sinuses, indicating that the detected tumor was primitive, and extended to the surrounding two sinuses (Figures 4A–C). Histopathological examination of a hematoxylin and eosin (H&E)-stained biopsy specimen revealed a single layer of uniform columnar cells with an eosinophilic cytoplasm, with rare mitotic figures forming a tubular growth that infiltrated the underlying stroma (Figures 5A,B). Immunohistochemistry revealed that the tumor cells were immunoreactive with antibodies against cytokeratin 7 (CK7) and p16 and were not immunoreactive with antibodies against cytokeratin 20 (CK20), CDX2, or MUC2 (Figures 5C–F). Based on these radiological and histological examinations, the sinonasal tumor was diagnosed as LG non-ITAC (T4aN0M0, Stage IVa).

The patient did not agree to be treated by radical resection (i.e., anterior craniofacial resection or orbital exenteration because of potential post-operative disfigurement and blindness. Rather, the patient selected chemotherapy concurrent with radiotherapy. We advised him to undergo carbon ion radiotherapy at the Research Center for Charged Particle Therapy, National Institute of Radiological Science (NIRS), Chiba, Japan; however, he declined the suggestion because the location was far from his home. After we presented him other options, the patient selected super-selective intra-arterial infusion of CDDP concurrent with radiotherapy (concurrent chemoradiotherapy) at our university as a radical treatment. After being explained about the risks and possible complications in response to concurrent chemoradiotherapy, we obtained his informed consent.

On September 14, 2011 (day 1), radiotherapy was started with administration by a megavoltage linear accelerator with a photon beam energy of 6 MeV. The radiotherapy was applied to the tumor and margins, with the radiation field also including the nasal cavity, the maxillary, ethmoid, and sphenoid sinuses, nasopharynx, base of the skull and hard palate. The radiotherapy was administered to the patient at a dose of 2.0 Gray (Gy), 5 times per week for 6 weeks, for a total radiotherapy dose of 60.0 Gy.

On day 9, selective angiography of the right maxillary artery was conducted by experienced radiologists using the Seldinger technique to evaluate the appropriate arteries supplying the tumor. CDDP at a dose of 150 mg/body was rapidly infused through a microcatheter for 20 min *via* an infusion pump. The tip of the microcatheter was placed in the feeding vessel of the tumor. For hydration, lactated Ringer's solution started 24 h prior to CDDP infusion was intravenously administered at 100 ml/h. Hydration was continued during and after CDDP infusion for 48 h. To neutralize the toxicity of the CDDP, an intravenous administration of sodium thiosulfate was started immediately before the CDDP infusion and was continued during the CDDP infusion. To minimize nausea and vomiting, a 5HT3-receptor antagonist was given to the patient for 4 days. By 4 days after the conclusion of radiotherapy (day 48), the patient had undergone 4 treatments with intra-arterial CDDP (1 injection every 2 weeks for 8 weeks).

During treatment, blood tests including hemograms, blood chemistry, and renal function were checked periodically. Acute toxicities related to the treatment were observed according to the National Cancer Institute-Common Toxicity Criteria (NCI-CTC). The patient experienced grade 1 non-hematologic side effects that consisted of ear pain (grade 1), mucositis oral (grade 1), nausea (grade 1), oral pain (grade 1), and alopecia (grade 1). There were no hematologic side effects.

At 10 days after CDDP infusion (day 19), the patient felt that his nasal obstruction had decreased. On day 60, endoscopy showed the disappearance of his right nasal polypoid mass filling the whole common meatus, and thereby, the right common meatus was well-open. In addition, only a bulge remained on the medial surface of the middle turbinate (Figure 1C). The mucous membranes of the nasal septum, the olfactory cleft, the superior and inferior turbinate, the middle meatus and the choana appeared to be normal (Figure 1D). On day 94, CT showed that opacification of the right nasal cavity had completely disappeared (Figures 2D–F). On day 95, T1- and T2-weighted MRI of the right ethmoid sinus confirmed a high signal intensity area (Figures 3C,D). On day 99, no uptake of FDG in the right sinonasal area was shown by PET (Figures 4D–F). These findings suggested that the chemotherapy concurrent with radiotherapy was markedly effective for this LG non-ITAC located in the right nasal cavity and paranasal sinus. Then, the patient indicated his preference to have a right transnasal endoscopic ethmoidectomy as salvage surgery to avoid functional and appearance problems as much as possible.

On day 128, right transnasal endoscopic ethmoidectomy was performed as salvage surgery. The middle and superior turbinates were completely removed along with the surrounding structures, which included the ethmoid sinus mucous membrane and the upper half of the nasal septum. No bone defects of the lamina papyracea, nasal septum or skull base were apparent. Histopathological examination revealed that the lesion had been replaced by granulation tissue with lymphocytic infiltration and hemosiderin-laden macrophages, and no viable tumor cells remained (Figures 5G,H). The histopathological findings also showed that intra-arterial CDDP administration concurrent with radiotherapy was an effective approach (i.e., complete remission had been achieved). At 7 years after concurrent chemoradiotherapy *followed by a salvage surgery*, the patient is alive without any local recurrence or regional or systemic metastasis. Thus, complete remission was clinically achieved in response to concurrent chemoradiotherapy followed by salvage endonasal endoscopic surgery, according to the new response criteria in solid tumors: Revised RECIST guideline (version 1.1).

## Discussion

Sinonasal non-ITAC is an adenocarcinoma without the histopathological characteristics of salivary or intestinal-type adenocarcinoma (5). Non-ITAC is additionally categorized into HG and LG types. LG non-ITAC is very uncommon. Thus, far, only two studies have reported the clinical and therapeutic aspects of LG non-ITAC (6, 12). We performed a search of the PubMed and Ichushi databases using the following search term, “sinonasal, low-grade, non-intestinal adenocarcinoma.” According to the results of our search, the present case represents the first report of a Japanese patient with LG non-ITAC.

The origins of LG non-ITAC have varied in previous reports. Stelow et al. reported that 64% of the LG non-ITACs arose in the nasal cavity, frequently in the middle turbinate, with 20% arising in the ethmoid sinus (5). Bhaijee et al. reported one case of LG non-ITAC originating in the nasal cavity (12). However, Bignami et al. reported that the tumor originated in the ethmoid sinus in 9 (39%) of 13 patients, with the tumor arising in the nasal cavity in the remaining cases (6). In our case, the ethmoid sinus was identified as the origin based on the results of examinations after chemoradiotherapy.

Histopathologically, LG non-ITAC tumors present varied architectural forms with exophytic papillae and tubular or glandular patterns having a LG cytology, round and uniform nuclei, and very rare mitotic figures (13). In our case, the histopathological examination of a biopsy specimen obtained before chemoradiotherapy showed tubular growth back-to-back as it infiltrated the underlying stroma, and a single-layer structure of uniform columnar cells with eosinophilic cytoplasm and rare mitotic figures. Based on these findings, it was classified as a LG tumor.

CK7 and CK20 are low molecular weight cytokeratins. The expression of CK7 was reported in a majority of epithelial neoplasms, with the exception of carcinoma arising from the colon, whereas CK20 positivity was seen in virtually all cases of colorectal carcinoma (13). As for CDX2, it is a cloned caudal-type homeobox gene that encodes a transcription factor with an important role in the proliferation and differentiation of intestinal epithelial cells (14). Thus, the immunohistological combination of CK20 and CDX2 positivity and CD7 negativity is currently used as a specific marker of adenocarcinomas such as ITAC (5, 15). Our case was CK7 positive and CK20 and CDX2 negative, which indicated the specific features of non-ITAC (5, 16).

P16 is a tumor suppressor protein inhibiting cyclin-dependent kinase 4A. p16 showing a strong and diffuse pattern of immunostaining is considered a highly sensitive surrogate marker for identifying HPV-driven tumors (17). Bishop et al. reported that p16-positive adenocarcinoma was found in the sinonasal tract in only 2 of 60 patients, in whom it was classified as HG non-ITAC (18). Thus, our case represents the first report of a Japanese patient with p16-positive LG non-ITAC.

The gold-standard treatment for primary sinonasal adenocarcinoma continues to be meticulous surgical tumor removal aiming at achieving free margins, followed by radiotherapy, and is indicated for the majority of patients (15). When the tumor involves the sphenoid sinus, total resection of the tumor is very difficult due to the topography and proximity to the internal carotid artery and optic nerves, the cavernous sinus and sella. Darouassi et al. described a case of poorly differentiated adenocarcinoma of the sphenoid sinus in which debulking surgery was performed followed by the radiotheraphy (19). The patient died 6 months after the therapy. Choussy et al. reported 418 patients with ethmoid sinus adenocarcinoma who showed a worse prognosis when the lesion reached the sphenoid sinus (7). Bignami et al. pointed out that LG non-ITAC showed a better prognosis compared with HG non-ITAC, and within the LG non-ITAC group, patients with T4a and T4b tumors had worse outcomes than those with T1 to T3 tumors (6). Approximately 25% of patients with LG non-ITAC develop recurrent disease, and 6% die from the tumor, usually as a result of the loss of local control (5). As the tumor extended into the sphenoid sinus, we recommended radical resection (i.e., anterior craniofacial resection and orbital exenteration).

The incorporation of induction chemotherapy in multimodality treatment of locally advanced sinonasal carcinoma has shown promising results (8, 20). Regarding adenocarcinoma, Licitra et al. performed a single-institution phase II study to investigate the effects of primary systemic chemotherapy with cisplatin, fluorouracil, and leucovorin (PEL combination) followed by surgery and radiotherapy in patients with paranasal cancer (21). They found that pathological complete remission was attained in 8 (16%) of 49 patients. As 37 (76%) of 49 patients had adenocarcinoma, they mentioned that sinonasal adenocarcinoma is a chemosensitive tumor. In sinonasal ITAC, Bossi et al. compared 5-year overall survival (5-year OS) and disease-free survival (5-year DFS) between 30 patients treated with surgery followed by radiotherapy (Group A) and 44 patients treated with induction chemotherapy with PLF combination followed by surgery and adjuvant radiotherapy (Group B) (22). The 5-year OS rate in Group B was 70%, whereas that in Group A was 42% (*p* = 0.041). The 5-year DFS rate in Group B was 66%, whereas that in Group A was 40%. In patients who received the PLF combination as induction chemotherapy, cardiovascular complications were reported to be a limiting toxicity (21). Apart from these isolated institutional experiences, the role of systemic chemotherapy in the induction or adjuvant setting in the management of both ITAC and non-ITAC has not been systematically investigated (23).

The administered dose of a drug and its concentration within a tumor are suggested to be critical factors related to the effectiveness of chemotherapy in most solid neoplasms (24). Based on this idea, intra-arterial infusion was proposed as an alternative route for systemic chemotherapy (11), with a goal of achieving higher drug concentrations in the tumor-bearing area and the possibility of lower recirculation and reduced toxicity (25). CDDP has an inhibiting effect on the repair of sublethal radiation damage (11). Thus, the intra-arterial infusion of cisplatin concurrent with radiation would seem to be an attractive therapeutic approach for obtaining local control with the potential for a long-term cure (11). Michael et al. reported the cases of 2 patients with locally advanced sinonasal adenocarcinoma treated with intra-arterial CDDP infusion (150 mg/m^2^, 4 times) concurrent with conventional fractionated radiation (total dose: 50 Gy) (26). Craniofacial resection was performed after concurrent chemoradiotherapy. One patient whose tumor was located in the nasal cavity and ethmoid sinus was alive with no evidence of disease at 46 months after the diagnosis, whereas the other patient whose tumor involved the sphenoid sinus, infratemporal fossa and anterior cranial fossa died 12 months after the diagnosis. We used super-selective intra-arterial CDDP infusion (150 mg/body) concurrent with conventional fractional radiotherapy for our patient with LG non-ITAC, which resulted in pathological and clinical complete remission and survival of the patient without any local recurrence or metastasis (regional or systemic) for 7 years after the course of chemoradiotherapy.

Surgery is still the primary treatment for sinonasal adenocarcinoma, and if the surgical margin is pathologically clear (11), can achieve a long-term cure. Chemoradiotherapy should be considered for those patients with unresectable local disease or who do not want to undergo surgery as a promising form of management (11). We described the case of a patient with primary, locally advanced sinonasal LG non-ITAC who was treated with intra-arterial CDDP administration concurrent with radiotherapy followed by salvage surgery. Although our procedure is essentially not a new treatment for sinonasal malignancies and other head and neck cancers, complete remission was ultimately achieved both histopathologically and clinically. Certainly, further clinical evaluation for a longer period is required to determine the clinical usefulness of concurrent chemoradiotherapy.

## Conclusion

We reported the first case of a Japanese patient in whom locally advanced sinonasal LG non-ITAC was successfully treated with concurrent chemoradiotherapy to achieve pathological and clinical complete remission. The administration of super-selective intra-arterial CDDP infusion concurrently with conventional fractionated radiotherapy *followed by salvage surgery* might be useful for the management of sinonasal adenocarcinoma, which is rare but more common in elderly patients.

## Data Availability Statement

The raw data supporting the conclusions of this article will be made available by the authors, without undue reservation.

## Ethics Statement

Written informed consent was obtained from the patient for the publication of any potentially identifiable images or data included in this article.

## Author Contributions

HTac, HTak, and HidS: conception and design, literature search, and writing the article. HTac, HTak, HidS, MF, KA, HK, NN, HirS, KN, SM, JI, and YM: obtain the images. All authors: critical revision and final approval of the article.

## Conflict of Interest

The authors declare that the research was conducted in the absence of any commercial or financial relationships that could be construed as a potential conflict of interest.
